# Insanity in Every-Day Practice

**Published:** 1919

**Authors:** 


**Insanity in Every-day Practice.** By E. G. Younger, M.D. Fourth Edition. Pp. x., 134. London: Baillière, Tindall & Cox. 1917. Price 5s. net.

A fourth edition requires little comment; its previous utility is obvious, and it is now improved by the addition of sections on Neurasthenia and on the Psycho-analysis of Freud. The latter, so much discussed of late years, has fallen into much disrepute from the exaggeration of the importance of questions of sex, which have been closely associated with the tedious process of psycho-analysis connected with the name of Professor Sigmund Freud, of Vienna. These views have much to commend them when the author's ideas on the sex element are modified.

